# What motivated medical students and residents to become radiation oncologists in Japan?—Questionnaire report by the radiotherapy promotion committee of JASTRO

**DOI:** 10.1093/jrr/rraa051

**Published:** 2020-07-22

**Authors:** Yuji Murakami, Shin-ei Noda, Yoshiomi Hatayama, Toshiya Maebayashi, Keiichi Jingu, Yasushi Nagata, Takashi Mizowaki

**Affiliations:** 1 Department of Radiation Oncology, Hiroshima University Hospital, Hiroshima, Japan; 2 Department of Radiation Oncology, Saitama medical university international medical center, Hidaka, Japan; 3 Department of Radiation Oncology, Hirosaki University School of medicine, Hirosaki, Japan; 4 Department of Radiology, Nihon University School of Medicine, Itabashi-ku, Japan; 5 Department of Radiation Oncology, Tohoku University Graduate School of Medicine, Sendai, Japan; 6 Department of Radiation Oncology & Image-applied Therapy, Kyoto University Graduate School of Medicine, Kyoto, Japan

**Keywords:** radiotherapy, medical students, radiation oncology education, questionnaire survey, clinical training, motivation to become radiation oncologist

## Abstract

This study aimed to clarify the motivations and timing of the decision to become radiation oncologists. Materials and methods: We conducted an online survey for new members of the Japanese Society for Radiation Oncology (JASTRO). Results: The response rate was 43.3%. Data of the 79 respondents who wanted to obtain a board-certification of JASTRO were analysed. We divided the respondents into two groups: Group A, those who entered a single radiation oncology department, and Group B, those who joined a radiology department in which the radiation oncology department and diagnostic radiology department were integrated. The most common period when respondents were most attracted to radiation oncology was “5th year of university” in Group A and “2nd year of junior residency” and “senior residency” in Group B. Furthermore, 79.5% of Group A and 40% of Group B chose periods before graduation from a university with a significant difference. The most common period when respondents made up their minds to become radiation oncologists was “2nd year of junior residency” in both groups. Internal medicine was the most common department to consider if they did not join the radiation oncology or radiology department. Conclusion: To increase the radiation oncologists, it is crucial to enhance clinical training in the fifth year of university for Group A and to continue an active approach to maintain interest in radiation oncology until the end of junior residency. In Group B facilities, it is desirable to provide undergraduates more opportunities to come in contact with radiation oncology.

## Introduction

The importance of radiotherapy has been increasingly recognised due to the recent development of high-precision radiotherapy and the promotion of a multidisciplinary treatment approach. The shortage of radiation oncologists is a long-standing problem in Japan. The number of radiation oncologists has recently increased due to the efforts of the Japanese Society for Radiation Oncology (JASTRO) and individual radiation oncologists. However, this is still not enough, and resolving this problem is an immediate task for JASTRO. JASTRO’s radiotherapy promotion committee conducts various activities related to recruiting medical students and residents and forecasting supply and demand. The mission of the working group for the promotion of radiation oncology education in this committee is to promote the enrolment of medical students and residents in radiation oncology departments. Regarding medical universities in Japan, there are two types of radiation oncology departments: one is the single radiation oncology department, and the other is the radiology department in which the radiation oncology and diagnostic radiology departments are integrated. In these different facilities, the timing and motivations for deciding to become a radiation oncologist may differ.

We, the working group for the promotion of radiation oncology education, conducted an online questionnaire survey of new JASTRO members in order to clarify the motives and timing of decisions that lead to them becoming radiation oncologists and to help the acquisition of radiation oncologists at each university hospital.

## Materials and methods

The subjects of the survey were new members of JASTRO who joined between April 2017 and June 2019, and who responded that they wanted to obtain a board-certification from JASTRO. In September 2019, a request to respond to an Internet-based survey created using Google Forms was e-mailed directly to all 208 new members. This survey included questions about the factors and timing that led to their emergence as radiation oncologists. The contents of the questionnaire are shown in Tables 1 and 2. We divided the respondents into two groups: Group A, those who entered a single radiation oncology department, and Group B, those who joined a radiology department where the radiation oncology department and diagnostic radiology department were integrated.

**Table 1:** Profiles of respondents

		All (n = 79)		Group A (n = 44)		Group B (n = 35)		Group A vs. B
		n	%	n	%	n	%	p
Gender								
	Men	58	73.4	32	72.7	26	74.3	n.s.
	Women	21	26.6	12	27.3	9	25.7	
Age								
	26–30	43	54.4	28	63.6	15	42.9	n.s.
	31–40	26	32.9	13	29.5	13	37.1	
	over 40	4	5.1	0	0.0	4	11.4	
	Unanswered	6	7.6	3	6.8	3	8.6	
Prefectures of graduated and joined university								
	Same	47	59.5	25	56.8	22	62.9	n.s.
	Different	32	40.5	19	43.2	13	37.1	
Doctor’s license acquisition year								
	2015–2017	65	82.3	40	90.9	25	71.4	0.03
	Until 2014	14	17.7	4	9.1	10	28.6	

**Table 2:** 

		All (n = 79)		Group A (n = 44)		Group B (n = 35)		Group A vs. B
		n	%	n	%	n	%	p
Q1: When did the respondents become most attracted to RO?								
	Before admission to university	7	8.9	4	9.1	3	8.6	n.s.
	1st-2nd year of university	3	3.8	3	6.8	0	0.0	n.s.
	3rd year of university	1	1.3	1	2.3	0	0.0	n.s.
	4th year of university	6	7.6	3	6.8	3	8.6	n.s.
	5th year of university	19	24.1	16	36.4	3	8.6	<0.01
	6th year of university	13	16.5	8	18.2	5	14.3	n.s.
	1st year of junior residency	7	8.9	2	4.5	5	14.3	n.s.
	2nd year of junior residency	12	15.2	4	9.1	8	22.9	n.s.
	Senior residency or after	11	13.9	3	6.8	8	22.9	0.04
Q2: Which educational programme led respondents to be attracted to RO?								
	General Lecture	6	7.6	3	6.8	3	8.6	n.s.
	Special Lecture by off-campus lecturer	1	1.3	1	2.3	0	0.0	n.s.
	Clinical training at 5th year of university	14	17.7	10	22.7	4	11.4	n.s.
	Clinical training at 6th year of university	17	21.5	9	20.5	8	22.9	n.s.
	Junior residency of RO department	13	16.5	7	15.9	6	17.1	n.s.
	Junior residency at other department	5	6.3	1	2.3	4	11.4	n.s.
	Senior residency of RO department	6	7.6	0	0	6	17.1	n.s.
	Senior residency at other department	1	1.3	NA	NA	1	2.9	NA
	None	4	5.1	4	9.1	0	0.0	n.s.
	Other	6	7.6	4	9.1	2	5.7	n.s.
	Unanswered	6	7.6	5	11.4	1	2.9	n.s.
Q3: When did the respondents set up their mind to become a radiation oncologist?								
	Before admission to medical university	1	1.3	1	2.3	0	0.0	n.s.
	1st-2nd year of university	1	1.3	1	2.3	0	0.0	n.s.
	3rd year of university	0	0	0	0.0	0	0.0	n.s.
	4th year of university	1	1.3	1	2.3	0	0.0	n.s.
	5th year of university	9	11.4	5	11.4	4	11.4	n.s.
	6th year of university	8	10.1	3	6.8	5	14.3	n.s.
	1st year of junior residency	6	7.6	3	6.8	3	8.6	n.s.
	2nd year of junior residency	38	48.1	26	59.1	12	34.3	0.02
	Senior residency or after	14	17.7	3	6.8	11	31.4	<0.01
	Other	1	1.3	1	2.3	0	0.0	n.s.
Q4. What factors inspired the respondents to become a radiation oncologist?								
	Treating cancer anywhere in the body	41	51.9	25	56.8	16	45.7	n.s.
	Organ preservation treatment	48	60.8	29	65.9	19	54.3	n.s.
	Treatment planning	36	45.6	22	50.0	14	40.0	n.s.
	Treatment efficacy by radiotherapy	44	55.7	26	59.1	18	51.4	n.s.
	Precision radiotherapy	23	29.1	15	34.1	8	22.9	n.s.
	Brachytherapy	2	2.5	1	2.3	1	2.9	n.s.
	Particle therapy	14	17.7	8	18.2	6	17.1	n.s.
	Nuclear medicine treatment	2	2.5	1	2.3	1	2.9	n.s.
	Radiation physics	6	7.6	4	9.1	2	5.7	n.s.
	Radiation biology	4	5.1	3	6.8	1	2.9	n.s.
	Future prospects of radiotherapy	46	58.2	25	56.8	21	60.0	n.s.
	Multidisciplinary team	9	11.4	5	11.4	4	11.4	n.s.
	Quality of life as a doctor	41	51.9	23	52.3	15	42.9	n.s.
	Others	10	12.7	4	9.1	6	17.1	n.s.
Q5. Why did respondents choose the university they joined?								
	It was their university of graduation	41	51.9	20	45.5	21	60.0	n.s.
	It was in hometown	29	36.7	17	38.6	12	34.3	n.s.
	It was in the area where respondents wanted to live	15	19.0	6	13.6	9	25.7	n.s.
	Good atmosphere	36	45.6	24	54.5	12	34.3	n.s.
	There was a doctor respondents could respect	25	31.6	19	43.2	6	17.1	0.01
	There was a close doctor (such as senior)	10	12.7	5	11.4	5	14.3	n.s.
	There was a doctor who became a role model	10	12.7	7	15.9	3	8.6	n.s.
	Briefing session of the department	8	10.1	6	13.6	2	5.7	n.s.
	A dinner party	11	13.9	9	20.5	2	5.7	n.s.
	Impact of young doctors	11	13.9	9	20.5	2	5.7	n.s.
	Impact of senior doctors	12	15.2	9	20.5	3	8.6	n.s.
	Impact of the professor	13	16.5	12	27.3	1	2.9	<0.01
	There were many doctors	18	22.8	15	34.1	3	8.6	<0.01
	There were few doctors	5	6.3	4	9.1	1	2.9	n.s.
	There are many related hospitals	9	11.4	8	18.2	1	2.9	0.03
	Can study abroad	6	7.6	5	11.4	1	2.9	n.s.
	Others	8	10.1	5	11.4	3	8.6	n.s.
Q6. Which other department did respondents consider if they did not join the RO or Radiology department?								
	Internal medicine	30	38.0	17	38.6	13	37.1	n.s.
	None (RO only)	11	13.9	6	13.6	5	14.3	NA
	Diagnostic radiology	5	6.3	5	11.4	NA	NA	n.s.
	Anaesthesiology	4	5.1	2	4.5	2	5.7	n.s.
	Surgery	4	5.1	3	6.8	1	2.9	n.s.
	Psychiatry	4	5.1	2	4.5	2	5.7	n.s.
	Paediatrics	3	3.8	0	0.0	3	8.6	n.s.
	Pathology	3	3.8	0	0.0	3	8.6	n.s.
	Neurology	3	3.8	1	2.3	2	5.7	n.s.
	Ophthalmology	2	2.5	2	4.5	0	0.0	n.s.
	Otorhinolaryngology	2	2.5	2	4.5	0	0.0	n.s.
	Orthopaedics	2	2.5	2	4.5	0	0.0	n.s.
	Neurosurgery	1	1.3	0	0.0	1	2.9	n.s.
	Urology	1	1.3	1	2.3	0	0.0	n.s.
	Dermatology	1	1.3	1	2.3	0	0.0	n.s.
	Plastic surgery	1	1.3	0	0	1	2.9	n.s.
	Rehabilitation	1	1.3	0	0	1	2.9	n.s.
	Others	1	1.3	0	0	1	2.9	n.s.
Q7. Opinions for JASTRO to increase radiation oncologists (Free comments)								

Abbreviations: RO = radiation oncology, JASTRO = Japanese Society for Radiation Oncology, NA = not applicable, n.s. = not significant

Regarding medical education in Japan, we receive six years of medical education at a university medical school and then take the national exam to become a licensed physician. Once licensed, for two years we receive initial training as junior residents and then join the respective departments. Upon admission to each department, we train for about three years as senior residents and become board-certified.

## Results

### Profiles of respondents (Table 1)

Of the 208 members, 90 responded to the questionnaire, with a response rate of 43.3%. Of these 90, 79 responded that they wanted to obtain a board-certification from JASTRO. We analysed the data of these 79 respondents. There were 21 (26.6%) women and 58 (73.4%) men. Regarding age, 43 (54.4%) were 26–30 years old, 26 (32.9%) were 31–40 years old, 4 (5.1%) were over 40 years old, and 6 (7.6%) did not answer. Prefectures where the university of graduation was located and prefectures where the university hospital was located were the same for 43 (54.4%) respondents, different for 32 (40.5%), and 4 (5.1%) did not answer. Sixty-five (82.3%) obtained medical doctor licenses from 2015 to 2017 and 14 (17.7%) before 2014. Forty-four (55.7%) respondents were in Group A and 35 (44.3%) were in Group B. Compared to Group B, the time between doctors’ licensing and joining JASTRO was significantly shorter for Group A (p = 0.03).

### Answers to questionnaire items (Table 2)

#### Q1: When did the respondents become most attracted to radiation oncology?

In Group A, “5th year of university” was the most common answer, accounting for 36.4%, followed by “6th year of university”, accounting for 18.2%. Group A chose “5th year of university” significantly more than Group B (p < 0.01). In Group B, “2nd year of junior residency” and “senior residency”’ were the most common answers, accounting for 22.9%. Group B chose “senior residency” significantly more than Group A (p = 0.04). Thirty-five (79.5%) respondents of Group A and 14 (40%) of Group B chose periods before graduation from a university, with a significant difference between the two groups (p < 0.001). Of note, 4 (9.1%) participants of Group A and 3 (8.6%) of Group B had been interested in radiation oncology before entering university.

#### Q2: Which educational programme led respondents to be attracted to radiation oncology?

In Group A, “clinical training at 5th year of university” was the most common answer, accounting for 22.7%, followed by “clinical training at 6th year of university”, accounting for 20.5%. In Group B, “clinical training at 6th year of university” was the most common answer, accounting for 22.9%, followed by “junior residency of radiation oncology department” and “senior residency of the radiation oncology department”, accounting for 17.1%.

#### Q3: When did the respondents set up their mind to become a radiation oncologist?

In Group A, “2nd year of junior residency” was the most common answer, accounting for 59.1%, followed by “5th year of university” accounting for 11.4%. Group A chose “2nd year of junior residency” significantly more than Group B (p = 0.02). In Group B, “2nd year of junior residency” was the most common answer, accounting for 34.3%, followed by “senior residency or after,” accounting for 31.4%. Group B chose “senior residency or after” significantly more than Group A (p < 0.01).

#### Q4: What factors inspired the respondents to become radiation oncologists?

Characteristics of radiotherapy, including “treating cancer anywhere in the body”, “organ preservation treatment”, “treatment planning”, and “treatment efficacy by radiotherapy” were the common answers in both groups. Moreover, “future prospects of radiotherapy” and “quality of life as a doctor” were also common answers. On the other hand, few respondents chose “brachytherapy”, “nuclear medicine treatment”, “radiation physics”, and “radiation biology”.

#### Q5: Why did respondents choose the university hospital they joined?

In Group A, “good atmosphere” was the most common answer, accounting for 54.5%, followed by “their university of graduation”, accounting for 45.5%, and “there was a doctor who the respondents could respect”, accounting for 43.2%. In Group B, “their graduated university” was the most common answer, accounting for 60%, followed by “in the respondent’s hometown” and “good atmosphere”, accounting for 34.3%. Group A chose “there was a doctor who the respondents could respect”, “impact of the professor”, “many staff”, and “many related hospitals”, significantly more than Group B (p = 0.01, < 0.01, < 0.01 and 0.03, respectively).

#### Q6: Which other department did respondents consider if they did not join the RO or Radiology department?

Internal medicine was the most common answer in both groups, accounting for 38.6% in Group A and 37.1% in Group B.

#### Q7: Opinions for JASTRO to increase radiation oncologists

There were many comments on this question. Of these, the following five were representative opinions. 1. Raise awareness of radiotherapy for medical students and the general public, 2. Enhance radiation oncology education at medical universities, 3. Establish single radiation oncology departments, 4. Create a good atmosphere in radiation oncology departments, and 5. Introduce newest treatment equipment.

## Discussion

The shortage of radiation oncologists in Japan has long been of significant concern. According to JASTRO’s biennial nationwide survey of radiation treatment facilities, the average number of board-certified radiation oncologists per surveyed facility was 0.76 (531 oncologists in 700 facilities) in 2009 and 1.22 (899 oncologists in 737 facilities) in 2015 [1, 2]. With the efforts of JASTRO and individual radiation oncologists, the number of radiation oncologists has been increasing, but this is still far from enough. In addition, advances in radiotherapy technology have expanded the use of high-precision radiotherapies such as intensity-modulated radiotherapy, stereotactic body radiotherapy, image-guided radiotherapy, and respiratory-gated radiotherapy. The administration of these high-precision radiation treatments requires excessive, long hours of work, which has led to a shortage of human resources. Therefore, resolving the shortage of radiation oncologists is a significant issue that must be addressed by JASTRO.

In Japan, radiation oncology departments and diagnostic radiology departments initially belonged to the same radiology department. In recent years, radiation oncology departments have separated from radiology departments, and approximately 40% of university hospitals currently providing radiotherapy have single radiation oncology departments. Therefore, in this study, we divided respondents into two groups: Group A, those who entered an independent radiation oncology department, and Group B, those who joined a radiology department in which the radiation oncology department and diagnostic radiology department were integrated.

Barton et al. reported that learning clinical oncology through experience and rotations has been superior to a lecture-based approach [3, 4]. This survey showed that in Group A, clinical training in their 5th year of university was the educational programme that most attracted respondents to radiation oncology. Therefore, it seems crucial to conduct programmes focusing on education and recruitment for medical students at this period. Moreover, focusing on clinical training appears to be more attractive to medical students than systematic lectures. On the other hand, the decision to become a radiation oncologist was most common during the second year of junior residency. Group A requires a meticulous and continuous approach to maintaining interest in radiation oncology until the end of the junior residency period.

In Group B, the period from the 2nd year of junior residency to senior residency training in radiation oncology was the most common period that attracted respondents to radiation oncology. Compared to Group A, Group B was significantly less interested in radiation oncology during the undergraduate period (p < 0.001). This may be due to the potentially limited number of lectures and clinical training on radiation oncology, as lectures and clinical training at Group B facilities are shared between diagnostic radiology and radiation oncology. In the review of the literature about teaching radiation oncology to medical undergraduates, it is suggested that for recruiting medical students to the radiation oncology department, teaching radiation oncology should begin early in the undergraduate period and should be mandatory for all students [5]. In Group B facilities, it is desirable to provide undergraduates more opportunities to come in contact with radiation oncology. To that end, the independence of the radiation oncology department is an important issue. In the “Basic Plan for Promotion of Cancer Control” formulated by the Japanese government, the promotion of radiotherapy is listed as essential in cancer treatment. It also states that as a measure to be taken, universities should strive to establish a specialised and organ-specific education system for cancer treatment (for example, “clinical oncology courses” or “radiation oncology courses”) [6]. The number of single radiation oncology departments has increased over time, but further increases are desired.

Seven of the 79 respondents had been interested in radiation oncology before entering university. This suggests that they had gained interesting information regarding radiotherapy before they entered university. With the efforts of JASTRO and individual radiation oncologists, the awareness of radiation oncology is indeed increasing. However, a relatively large number of respondents answered, “raise awareness of radiotherapy for medical students and the general public”, for the question regarding “opinions for JASTRO to increase radiation oncologists”. Therefore, we need more active education and activities to raise awareness of radiotherapy not only for medical students but also for the general public.

Common factors that inspired respondents to become radiation oncologists included characteristics of radiotherapy, such as “treating cancer anywhere in the body”, “organ preservation treatment”, “treatment efficacy”, “treatment planning”, and so on. Also, more than half of the respondents chose “future prospects of radiotherapy” and “quality of life as a doctor”. Thus, we had better also emphasise these factors while educating about radiation oncology.

Regarding the factors in which respondents chose the university they joined, geographical reasons were dominant, but the “good atmosphere” of the department was selected most often in Group A and second-most in Group B. Moreover, the development of attractive human resources also seemed to be an essential factor.

The most common answer to the question of “Which other department did respondents consider if they did not join the radiation oncology or radiology department?” was internal medicine. With that in mind, radiation oncologists must teach the fun of radiation oncology over internal medicine. It is necessary to create a model curriculum for radiation oncology education that is available to all radiation oncologists and to build a standalone clinical practice rotation that can convey the interest of radiation oncology.

This study has several limitations. The response rate to the questionnaire was not high, at 43.3%, even though we requested responses to the questionnaire thrice and the deadline for answers was extended twice. One reason may be that the survey was anonymous. Therefore, the present sample may have comprised a relatively motivated cohort, whose responses may have differed from the opinions of a more general sample of candidates. In addition, this survey did not obtain sufficient information to analyse the geographic and institutional factors influencing the respondents. However, there are few reports on the motivation to become a radiation oncologist and, therefore, the results of the present study are considered highly significant. Regular surveys and comparisons over time are also important. In the next survey, we would like to devise a strategy to increase the response rate and to obtain more information.

In conclusion, to increase the number of radiation oncologists, in Group A, it is crucial to enhance clinical training in the fifth year of university and to continue an active approach to maintain interest in radiation oncology until the end of junior residency. In Group B facilities, it is desirable to provide undergraduates more opportunities to come in contact with radiation oncology.

## Presentation at a conference

We presented partial contents of this study at the 32^nd^ annual meeting of the Japanese Society for Radiation Oncology.
